# Antidepressants and Changes in Concentration of Endocannabinoids and *N*-Acylethanolamines in Rat Brain Structures

**DOI:** 10.1007/s12640-014-9465-0

**Published:** 2014-03-21

**Authors:** Irena Smaga, Beata Bystrowska, Dawid Gawliński, Bartosz Pomierny, Piotr Stankowicz, Małgorzata Filip

**Affiliations:** Department of Toxicology, Faculty of Pharmacy, College of Medicum, Jagiellonian University, 9, Medyczna Street, 30-688 Kraków, Poland

**Keywords:** Endocannabinoid, *N*-Acylethanolamine, Depression, Antidepressant

## Abstract

The endocannabinoid (eCB) system has recently been implicated in both the pathogenesis of depression and the action of antidepressants. Here, we investigated the effect of acutely or chronically administering antidepressants [imipramine (IMI) (15 mg/kg), escitalopram (ESC) (10 mg/kg), and tianeptine (10 mg/kg)] on the levels of both eCBs [anandamide (AEA) and 2-arachidonoylglycerol (2-AG)] and *N*-acylethanolamines (NAEs) [palmitoylethanolamide (PEA) and oleoylethanolamide (OEA)] in various rat brain regions. We also examined the ability of the acute and chronic administration of *N*-acetylcysteine (NAC) (a mucolytic drug; 100 mg/kg) or URB597 (a fatty acid amide hydrolase inhibitor; 0.3 mg/kg), which have both elicited antidepressant activity in preclinical studies, to affect eCB and NAE levels. Next, we determined whether the observed effects are stable 10 days after the chronic administration of these drugs was halted. We report that the chronic administration of all investigated drugs increased AEA levels in the hippocampus and also increased both AEA and 2-AG levels in the dorsal striatum. NAE levels in limbic regions also increased after treatment with IMI (PEA/OEA), ESC (PEA), and NAC (PEA/OEA). Removing chronic ESC treatment for 10 days affected eCB and NAE levels in the frontal cortex, hippocampus, dorsal striatum, and cerebellum, while a similar tianeptine-free period enhanced accumbal NAE levels. All other drugs maintained their effects after the 10-day washout period. Therefore, the eCB system appears to play a significant role in the mechanism of action of clinically effective and potential antidepressants and may serve as a target for drug design and discovery.

## Introduction

Depression is the leading cause of disability and the 4th highest contributor to the global disease burden in the twenty-first century. Despite the existence of several preclinical and clinical studies, the pathophysiology of this brain disorder remains unclear. In addition, currently prescribed antidepressants are often therapeutically inadequate in many patients. Although the role of stress, infectious agents, and genetic influence in depression has been well documented, the cause(s) of depression have not yet been completely elucidated. Previous attempts to identify the pathomechanism of depression have relied on the mechanism of action of antidepressants; however, the psychopharmacology of these drugs is also poorly understood. In the clinic, several antidepressants with different mechanisms of action are commonly used, which suggests that the specific interaction of the drug with its target is not responsible for its therapeutic efficacy; instead, there is likely a secondary effect that is important. The endocannabinoid (eCB) system is involved in modulating emotional responses, memory and learning, and several previous studies have implicated this system in the pathogenesis of depression. Some possible mechanisms of action include relocalizing CB_1_ receptors (among the limbic system, the reward system and midbrain monoaminergic nuclei), modulating monoaminergic transmission (through noradrenaline (NA), serotonin (5-HT), dopamine (DA), γ-aminobutyric acid (GABA), and glutamate), inhibiting the activation of the stress axis and promoting neuroplasticity in the brain (Micale et al. 2013). Eliminating CB_1_ receptors in mice results in a phenotype that closely resembles the symptoms of severe, typical depression, while blocking CB_1_ receptor induces a melancholic depression (Sanchis-Segura et al. 2004; Aso et al. 2008; Steiner et al. 2008; Mikics et al. 2009). In human clinical trials, patients who received the CB_1_ receptor antagonist rimonabant (SR141716A) to treat obesity also experienced symptoms of anxiety and depression (Christensen et al. 2007).

Several studies have also suggested that facilitating the eCB system by inhibiting fatty acid amide hydrolase (FAAH) URB597 promotes a positive mood and possibly exerts antidepressant-like behavioral responses in rodents (Rutkowska and Jachimczuk 2004; Gobbi et al. 2005; Hill and Gorzalka 2005; Jiang et al. 2005; Bambico et al. 2007; Naidu et al. 2007; Adamczyk et al. 2008; Realini et al. 2011). Other recent studies have implicated the eCB system in behavioral changes following antidepressant drug treatment (Hill et al. 2006, 2008b, c; Rodriguez-Gaztelumendi et al. 2009; Mato et al. 2010).

The goals of this study were twofold. First, we set out to determine the effect of chronic or acute administration of antidepressant drugs on biomarkers in the eCB system by analyzing eCB and eCB-like molecules in the rat brain either 24 h later or after a 10-day drug-free period following chronic drug administration. Second, we wanted to characterize the common adaptive changes that follow the administration of these antidepressant drugs. We first focused on determining whether the acute or chronic administration of antidepressants affected the levels of eCBs [anandamide (AEA) and 2-arachidonoylglycerol (2-AG)] or *N*-acylethanolamines (NAEs) [oleoylethanolamide (OEA) and palmitoylethanolamide (PEA)]. A subsequent 10-day drug-free period was implemented to determine whether the effects of these drugs on eCB/NAE levels are maintained after the treatment is discontinued. We selected those antidepressants that are most commonly prescribed by doctors, including imipramine (IMI, a NA and 5-HT reuptake inhibitor), escitalopram (ESC, a selective 5-HT reuptake inhibitor), and tianeptine (TIA, a selective 5-HT reuptake enhancer) along with drugs in which antidepressant activity has been more recently demonstrated in preclinical research, including URB597 (a FAAH inhibitor) (Gobbi et al. 2005; Adamczyk et al. 2008) and *N*-acetylcysteine (NAC, a mucolytic drug and a putative precursor of the primary tissue antioxidant glutathione) (Ferreira et al. 2008; Smaga et al. 2012). Previous studies have demonstrated that URB597, a selective inhibitor of the enzyme (FAAH) that catalyzes the intracellular hydrolysis of eCBs, can exert potent antidepressant-like effects in the mouse tail-suspension test (TST) and the rat forced-swim test (FST) that are comparable to those seen after IMI treatment (Gobbi et al. 2005; Adamczyk et al. 2008). The chronic administration of NAC was also found to exert an antidepressant-like effect in a dose-dependent manner in rats, which was demonstrated by the reduction in immobility time in the FST (Ferreira et al. 2008). In our study, bulbectomized rats exhibited a similar reduction, which was associated with the reinforcement of brain antioxidant defense mechanisms (Smaga et al. 2012).

## Materials and Methods

### Animals

The experiments were performed on male Wistar rats (250–300 g). The animals were kept on normal day–night cycle, at 22 ± 2 °C with access to food and water ad libitum. All experiments were carried out in accordance with the National Institutes of *Health Guide for the Care and Use of Laboratory Animals* and with approval of the Bioethics Commission as compliant with the Polish Law (21 August 1997). *N* = 8 rats/group.

### Drugs

The following drugs were used: imipramine hydrochloride (IMI; Sigma Aldrich, USA), escitalopram oxalate (ESC; Lundbeck, Denmark), tianeptine sodium (TIA; Anpharm, Poland), *N*-acetylcysteine (NAC; Sigma Aldrich, USA) and cyclohexylcarbamic acid 3-carbamoylbiphenyl-3-yl ester (URB597, Sigma Aldrich, USA). IMI, ESC, TIA, and NAC were dissolved in sterile 0.9 % NaCl (pH of a NAC and ESC solution has been neutralized with 10 % NaOH solution). URB597 was dissolved in 2–3 drops of ethanol and diluted as required in a 1 % aqueous solution Tween 80. Drugs were given once per day between 9:00 and 12:00 *ip* acutely or chronically (14 days), in addition, single dose of URB597 (0.3 mg/kg) was injected 2 h before decapitation of rats (*N* = 6 rats) to control the method of eCBs/NAEs determination (Table 1). The injection volume was 1 ml/kg of body weight. The doses for drugs were chosen based on effective doses used in our previous behavioral observations: NAC (100 mg/kg) (Smaga et al. 2012) and URB597 (Adamczyk et al. 2008) as well as in other literature findings on IMI (15 mg/kg) (Tokita et al. 2012), ESC (10 mg/kg) (Reed et al. 2009), and TIA (10 mg/kg) (Whitton et al. 1991).

**Table 1:** Experimental protocol

1–13 days	14 day	
Single administration		
Vehicle	IMI	Decapitation—at 24 h after last injection
Vehicle	ESC	
Vehicle	Tianeptine	
Vehicle	*N*-Acetylcysteine	
Vehicle	URB597	
–	URB597	Decapitation—at 2 h after injection
Chronic administration		
IMI	IMI	
ESC	ESC	Decapitation—at 24 h after last injection
Tianeptine	Tianeptine	
*N*-Acetylcysteine	*N*-Acetylcysteine	
URB597	URB597	
Chronic administration with 10-day washout period		
IMI	IMI	Decapitation—at 10 days after last injection
ESC	ESC	
Tianeptine	Tianeptine	
*N*-Acetylcysteine	*N*-Acetylcysteine	
URB597	URB597	

For each drug the control group of rats was generated by single or chronic administration of corresponding vehicle. *N* = 6–8 rats/group

### Brain Structures Isolation

2 h (single administration of URB597), 24 h (acute and chronic administration), or 10 days (washout period after 14-day chronic administration) after last administration rats were sacrificed through decapitation. Selected brain structures (i.e., the prefrontal cortex, frontal cortex, hippocampus, dorsal striatum, nucleus accumbens, and cerebellum) were isolated, immediately frozen on dry ice and stored at −80 °C. Tissues were dissected out according to The Rat Brain Atlas (Paxinos and Watson 1998).

### LC–MS/MS Analysis

#### Reagents

All chemical solvents and standards were of analytical grade. Standards of AEA, 2-AG, OEA, and PEA were obtained from Tocris (Bristol, United Kingdom), AEA-d_4_, 2-AG-d_5_, OEA-d_4_, and PEA-d_4_ from Cayman Chemical (USA), acetonitrile and chloroform from Merck (Darmstadt, Germany), methanol and formic acid from POCh (Katowice, Poland). Standards stock solutions were prepared in ethanol, except from 2-AG and 2-AG-d_5_ which were prepared in acetonitrile. All stock solutions were stored at −80 °C. Further dilutions were carried out appropriately in acetonitrile.

#### Lipid Extraction from Brain Tissue

The brain tissues were weighted and subjected to eCB and NAE extraction. Extraction was carried out by the modified methods of isolation of lipid compounds developed by Folch et al. (1957). Tissues were homogenized using sonificator (UP50H, Hielscher) in the ice-cold mixture of methanol and chloroform (1:2; v/v) in proportion 10 mg of wet tissue to 150 μl of solvent to quench any possible enzymatic reaction that may interfere with the analysis. Next, 150 μl of homogenate were mixed with 2 μl of internal standard (AEA-d_4_, concentration 10 μg/ml; 2-AG-d_5_, concentration 100 μg/ml; PEA-d_4_, OEA-d_4_, concentration 5 μg/ml), 250 μl of formic acid (pH 3.0; 0.2 M) and 1,500 μl of extraction mixture (methanol:chloroform; 1:2, v/v). The internal standard indicates analyte loss during sample work-up. Afterward, samples were vortexed for 30 s and centrifuged for 10 min at 2,000 rpm. Organic phases were collected and dried under a stream of nitrogen at 40 °C. The residue was dissolved in 40 μl of acetonitrile, and 10 μl of the reconstituted extract was injected into the LC–MS/MS system for quantitative analysis.

#### LC–MS/MS Conditions

LC was performed using an Agilent 1100 (Agilent Technologies, Waldbronn, Germany) LC system. Chromatographic separation was carried out with a Thermo Scientific BDS HYPERSIL C18 column (100 × 3 mm I.D., 3 μm particle size). The advance column, with precolumn (10 × 3 mm I.D., 3 μm particle size) set at 40 °C with a mobile phase flow rate of 0.3 ml/min. Gradient elution mobile phases were consisted of formic acid (0.02 M) in water (solvent A) and formic acid (0.02 M) in acetonitrile (solvent B). The gradient began initially at 0 % A during 1 min, increasing linearly to 90 % at 2 min, this was maintained for 2 min and then decreasing to 0 % at 6 min. Finally, last 4 min of analysis was kept at 100 % B. Sample temperature was maintained at 4 °C in the autosampler prior to analysis. A sample volume of 10 μl was injected into the analytical column for compound analysis.

MS/MS analyses were accomplished on an Applied Biosystems MDS Sciex (Concord, ON, Canada) API 2000 triple quadruple mass spectrometer equipped with an electrospray ionization (ESI) interface. ESI ionization was performed in the positive ionization mode. A standard polypropylene glycols solution (PPG standard) was used for instrument tuning and mass calibration at unit mass resolution according to the Applied Biosystems manual. The mass spectrometer was operated with a dwell time of 200 ms. To find the optimal parameters of ion path and ion source of the studied compound, the quantitative optimization was done by direct infusion of standards using a Hamilton syringe pump (Hamilton, Reno, NV, USA). Multiple reaction monitoring (MRM) mode of the dominant product ion for each eCB/NAE was realized using the optimal conditions. The ion source parameters were as follows: ion spray voltage (IS): 5,500 V; nebulizer gas (gas 1): 30 psi; turbo gas (gas 2): 10 psi; temperature of the heated nebulizer (TEM): 400 °C; curtain gas (CUR): 25 psi. Comparison of pair ion (precursor and product ion *m*/*z* values) and LC retention times with standards served to confirm the identification of eCB/NAE in the samples investigated. Ion pair was 348/62 for AEA, 379/287 for 2-AG, 326/62 for OEA, 300/62 for PEA, 352/66 for AEA-d_4_, 384/292 for 2-AG-d_5_, 330/66 for OEA-d_4_, and 304/66 for PEA-d_4_. Data acquisition and processing were accomplished using the Applied Biosystems Analyst version 1.4.2 software.

#### Calibration Curve and Quantification

eCB and NAE concentrations in samples were calculated using the calibration curve that was prepared on the same day and analyzed in the same analytical run. Calibration curves were constructed after the analysis of samples of brain tissues collected from naive rats. The homogenates were spiked with AEA, OEA, and PEA to the following concentration: blank, 0.1, 1, 10, 25, 50, 100 ng/g. Solutions used for 2-AG were: blank, 0.4, 1, 5, 10, 25, 50 μg/g. AEA-d_4_, 2-AG-d_5_, PEA-d_4_, OEA-d_4_ were used as the internal standard. These samples were analyzed according to the procedure described for sample preparation (“Lipid extraction from brain tissue” section).

### Statistical Analyses

All data were expressed as means (±SEM). Statistical analyses were performed with either Student’s *t* test or one-way analysis of variance (ANOVA), followed by Dunnett’s test to analyze differences between group means. *p* < 0.05 was considered statistically significant.

## Results

### Concentration of eCB in Rat Brain Structures

#### AEA

**Fig. 1:**
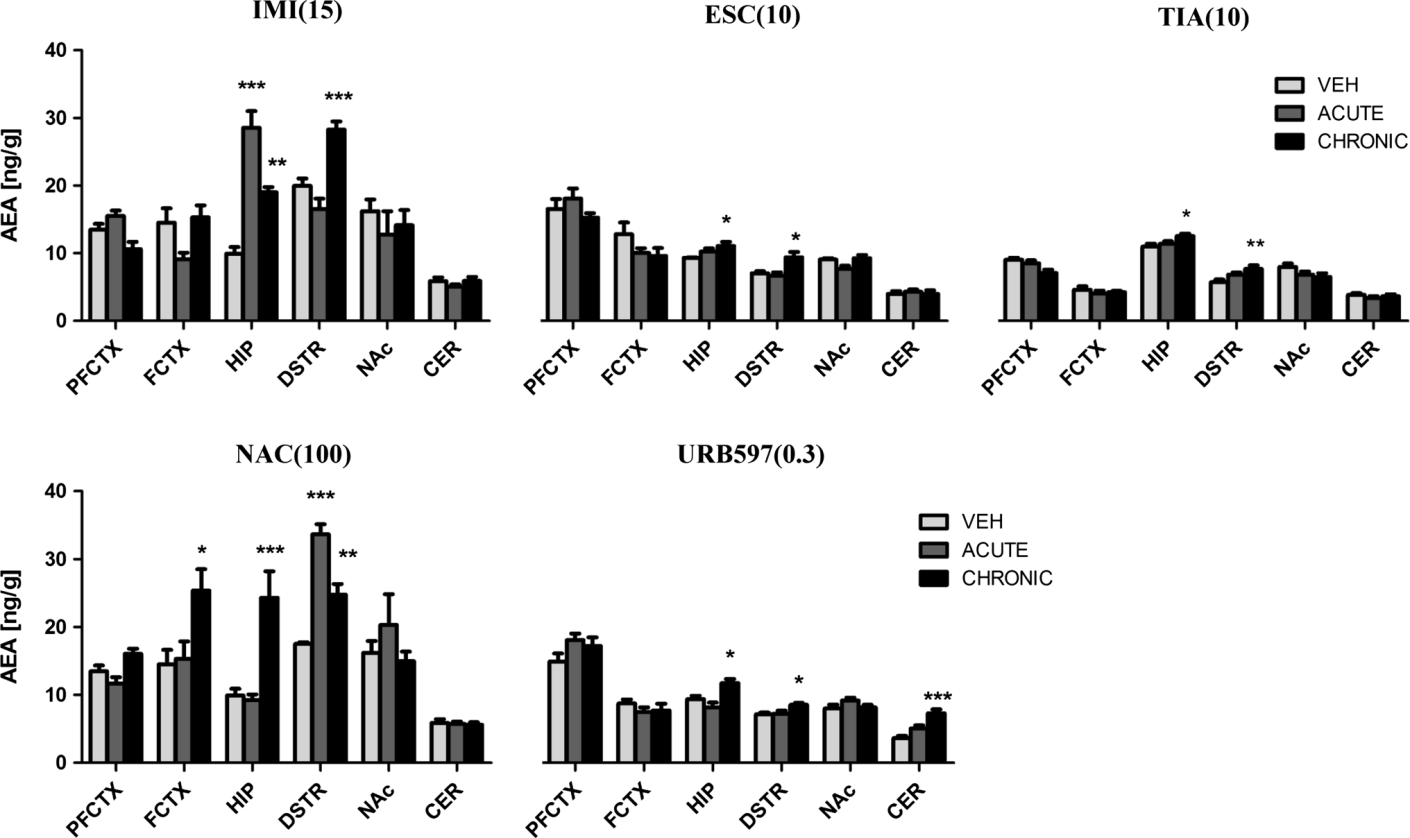
AEA levels in rat brain structures following acute and chronic drug/compound administration. *AEA* Anandamide, *IMI(15)* imipramine hydrochloride (15 mg/kg), *ESC(10)* escitalopram oxalate, *TIA(10)* tianeptine sodium, *NAC(100)* *N*-acetylcysteine, *URB597(0.3)* cyclohexylcarbamic acid 3-carbamoylbiphenyl-3-yl ester, *PFCTX* prefrontal cortex, *FCTX* frontal cortex, *HIP* hippocampus, *DSTR* dorsal striatum, *NAc* nucleus accumbens, *CER* cerebellum. All data are expressed as the mean ± SEM. *N* = 8 rats/group. *p < 0.05; **p < 0.01; ***p < 0.001 versus corresponding vehicle

**Fig. 2:**
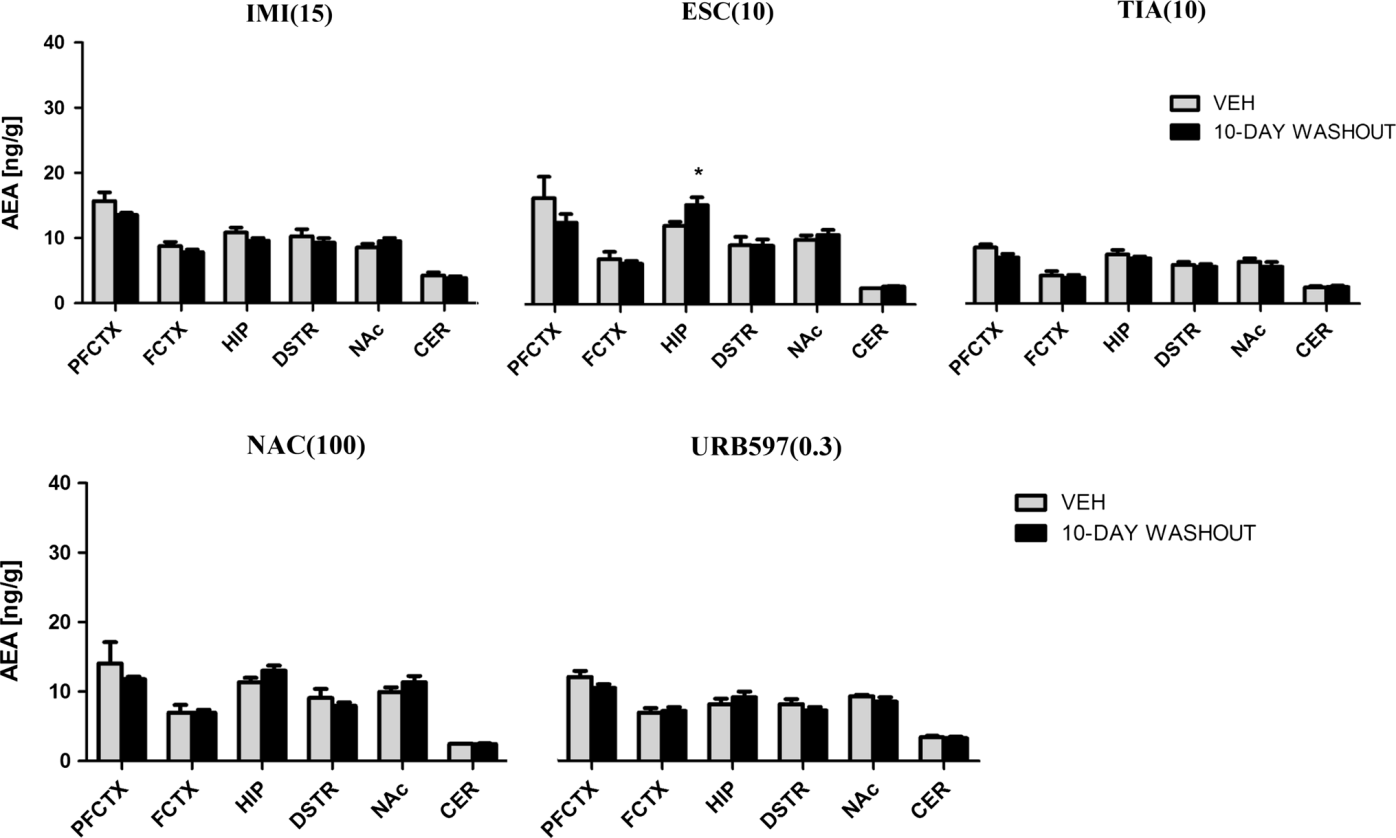
AEA levels in rat brain structures following chronic drug/compound administration and 10-day washout period. *AEA* anandamide, *IMI(15)* imipramine hydrochloride (15 mg/kg), *ESC(10)* escitalopram oxalate, *TIA(10)* tianeptine sodium, *NAC(100)* *N*-acetylcysteine, *URB597(0.3)* cyclohexylcarbamic acid 3-carbamoylbiphenyl-3-yl ester, *PFCTX* prefrontal cortex, *FCTX* frontal cortex, *HIP* hippocampus, *DSTR* dorsal striatum, *NAc* nucleus accumbens, *CER* cerebellum. All data are expressed as the mean ± SEM. *N* = 8 rats/group. *p < 0.05 versus corresponding vehicle

IMI (15 mg/kg) treatment caused the changes in the AEA levels in the hippocampus (*F*(2,21) = 34.29; *p* < 0.0001) and dorsal striatum (*F*(2,21) = 21.21; *p* < 0.0001). Post hoc analyses revealed the significant increase of AEA in the hippocampus (*p* < 0.001) after acute administration of IMI. After chronic administration of IMI, an increase of AEA levels was reported in the hippocampus (*p* < 0.01) and dorsal striatum (*p* < 0.001) (Fig. 1). A 10-day washout period after chronic treatment of IMI restored the levels of AEA to the levels of vehicle-treated animals in all structures (Fig. 2).

After ESC (10 mg/kg) treatment, the changes in the AEA levels were seen in the hippocampus (*F*(2,21) = 0.3888; *p* = 0.0366) and dorsal striatum (*F*(2,21) = 7.240; *p* = 0.0041). After chronic administration of ESC, an increase of AEA concentration was noted in the hippocampus (*p* < 0.05) and dorsal striatum (*p* < 0.05), while acute administration of ESC did not change the basal levels of AEA (Fig. 1). 10 days after the last administration, an increase of AEA levels was seen only in the hippocampus (*t* = 2.407, df = 14, *p* < 0.05) (Fig. 2).

TIA (10 mg/kg) evoked changes in the AEA concentration in the hippocampus (*F*(2,21) = 4.036; *p* = 0.0329) and dorsal striatum (*F*(2,21) = 5.703; *p* = 0.0105). Acute administration of TIA did not change AEA levels, whereas repeated daily injections of TIA resulted in an increase in the hippocampus (*p* < 0.05) and dorsal striatum (*p* < 0.01) (Fig. 1). A 10-day washout period after chronic treatment of TIA restored the levels of AEA to the levels of vehicle-treated animals in all structures (Fig. 2).

NAC (100 mg/kg) treatment resulted in changes of AEA levels in the frontal cortex (*F*(2,21) = 5.209; *p* = 0.0146), hippocampus (*F*(2,21) = 12.91; *p* = 0.0002) and dorsal striatum (*F*(2,21) = 37.10; *p* < 0.0001). Acute administration of NAC increased the AEA levels in the dorsal striatum (*p* < 0.001), while chronic administration of NAC increased the AEA levels in the frontal cortex (*p* < 0.05), hippocampus (*p* < 0.001), and dorsal striatum (*p* < 0.01) (Fig. 1). A 10-day washout period after chronic treatment of NAC restored the levels of AEA to the levels of vehicle-treated animals in all structures (Fig. 2).

Administration of URB597 (0.3 mg/kg) caused the changes in the AEA levels in the hippocampus (*F*(2,21) = 8.311; *p* = 0.0022), dorsal striatum (*F*(2,21) = 5.787; *p* = 0.01) and cerebellum (*F*(2,21) = 17.03; *p* < 0.0001). Chronic administration of URB597 evoked an increase of AEA levels in the hippocampus (*p* < 0.05), dorsal striatum (*p* < 0.05), and cerebellum (*p* < 0.001) (Fig. 1). Neither acute administration nor 10-day drug-free period changed the AEA levels in the examined rat brain structures (Fig. 2).

For comparison, the levels of AEA measured 2 h after single administration of URB597 increased in the hippocampus (*t* = 4.342, df = 10, *p* < 0.01), dorsal striatum (*t* = 3.172, df = 10, *p* < 0.01), and cerebellum (*t* = 4.515, df = 10, *p* < 0.01) (Table 2).

**Table 2:** Effects on the levels of eCBs in rat brain structures measured 2 h after single administration of URB597 (0.3 mg/kg)

	AEA		2-AG		PEA		OEA	
	VEH	URB597	VEH	URB597	VEH	URB597	VEH	URB597
PFCTX	4.763 ± 0.20	4.692 ± 0.18	2.236 ± 0.26	2.218 ± 0.42	94.65 ± 9.42	130.3 ± 15.60	83.86 ± 8.83	83.8 ± 5.66
FCTX	4.325 ± 0.73	4.519 ± 0.37	2.367 ± 0.32	2.159 ± 0.23	117.7 ± 15.21	107.2 ± 20.32	92.72 ± 6.62	90.43 ± 8.41
HIP	3.897 ± 0.19	6.318 ± 0.58**	2.929 ± 0.20	2.757 ± 0.17	114.2 ± 16.77	210.0 ± 22.29**	83.08 ± 10.72	118.2 ± 7.46*
DSTR	3.851 ± 0.16	4.703 ± 0.21**	2.799 ± 0.23	2.203 ± 0.22	139.0 ± 9.46	337.2 ± 20.14***	87.47 ± 4.57	193.3 ± 21.86***
NAc	4.998 ± 0.24	4.085 ± 0.36	2.772 ± 0.12	2.915 ± 0.26	116.8 ± 4.51	325.3 ± 25.68***	79.26 ± 4.29	166.0 ± 19.69**
CER	3.594 ± 0.10	5.592 ± 0.43**	2.232 ± 0.49	2.096 ± 0.24	97.45 ± 10.21	99.54 ± 9.67	98.98 ± 1.23	99.49 ± 0.77

All data are expressed as the mean ± SEM. *N* = 6 rats/group

*URB597(0.3)* Cyclohexylcarbamic acid 3-carbamoylbiphenyl-3-yl ester (0.3 mg/kg), *AEA* anandamide, *2-AG* 2-arachidonoylglycerol, *PEA* palmitoylethanolamide, *OEA* oleoylethanolamide

* *p* < 0.05; ** *p* < 0.01; *** *p* < 0.001 versus vehicle (VEH)

#### 2-AG

**Fig. 3:**
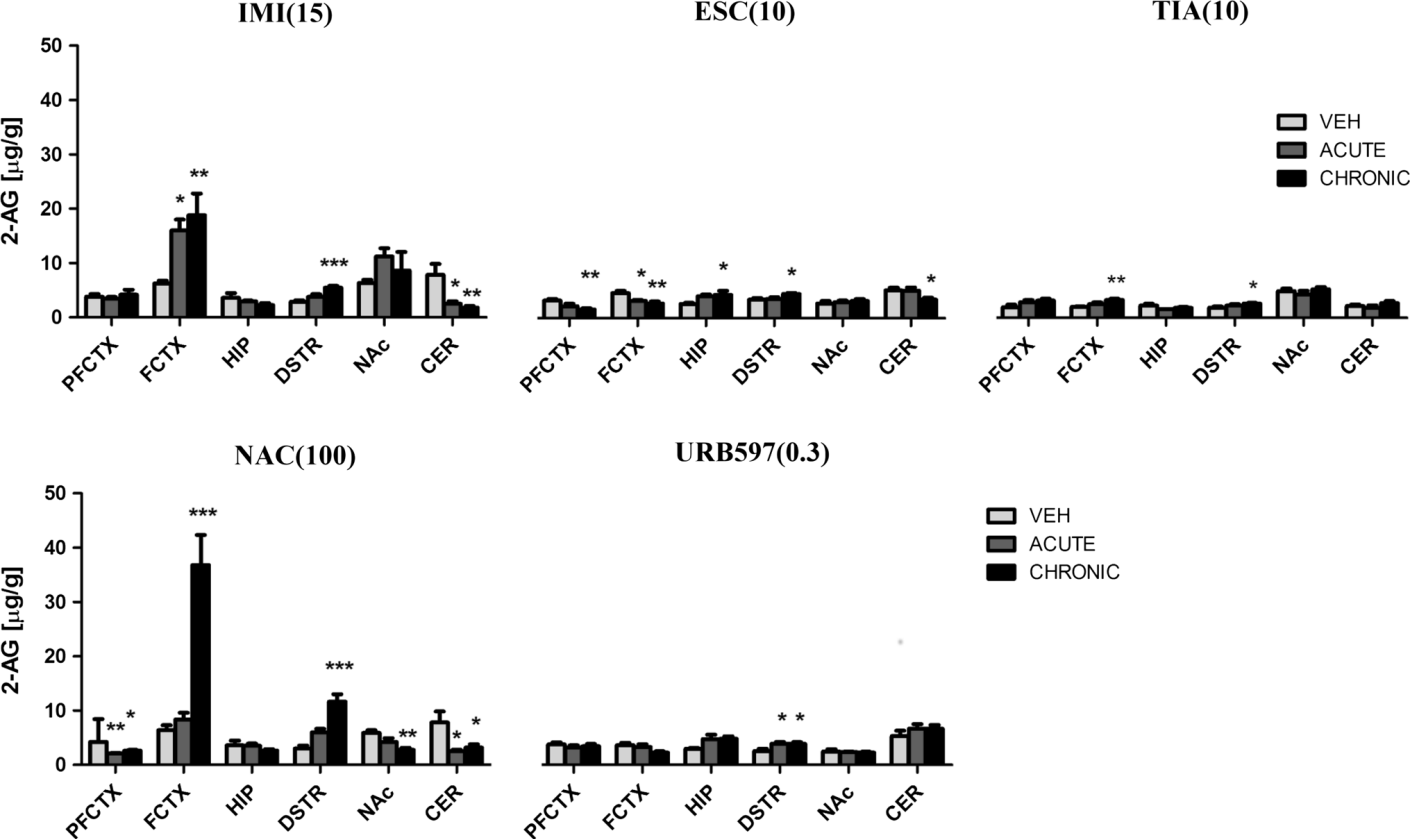
2-AG levels in rat brain structures following acute and chronic drug/compound administration. *2-AG* 2-Arachidonoylglycerol, *IMI(15)* imipramine hydrochloride (15 mg/kg), *ESC(10)* escitalopram oxalate, *TIA(10)* tianeptine sodium, *NAC(100)* *N*-acetylcysteine, *URB597(0.3)* cyclohexylcarbamic acid 3-carbamoylbiphenyl-3-yl ester, *PFCTX* prefrontal cortex, *FCTX* frontal cortex, *HIP* hippocampus, *DSTR* dorsal striatum, *NAc* nucleus accumbens, *CER* cerebellum. All data are expressed as the mean ± SEM. *N* = 8 rats/group. *p < 0.05; **p < 0.01; ***p < 0.001 versus corresponding vehicle

**Fig. 4:**
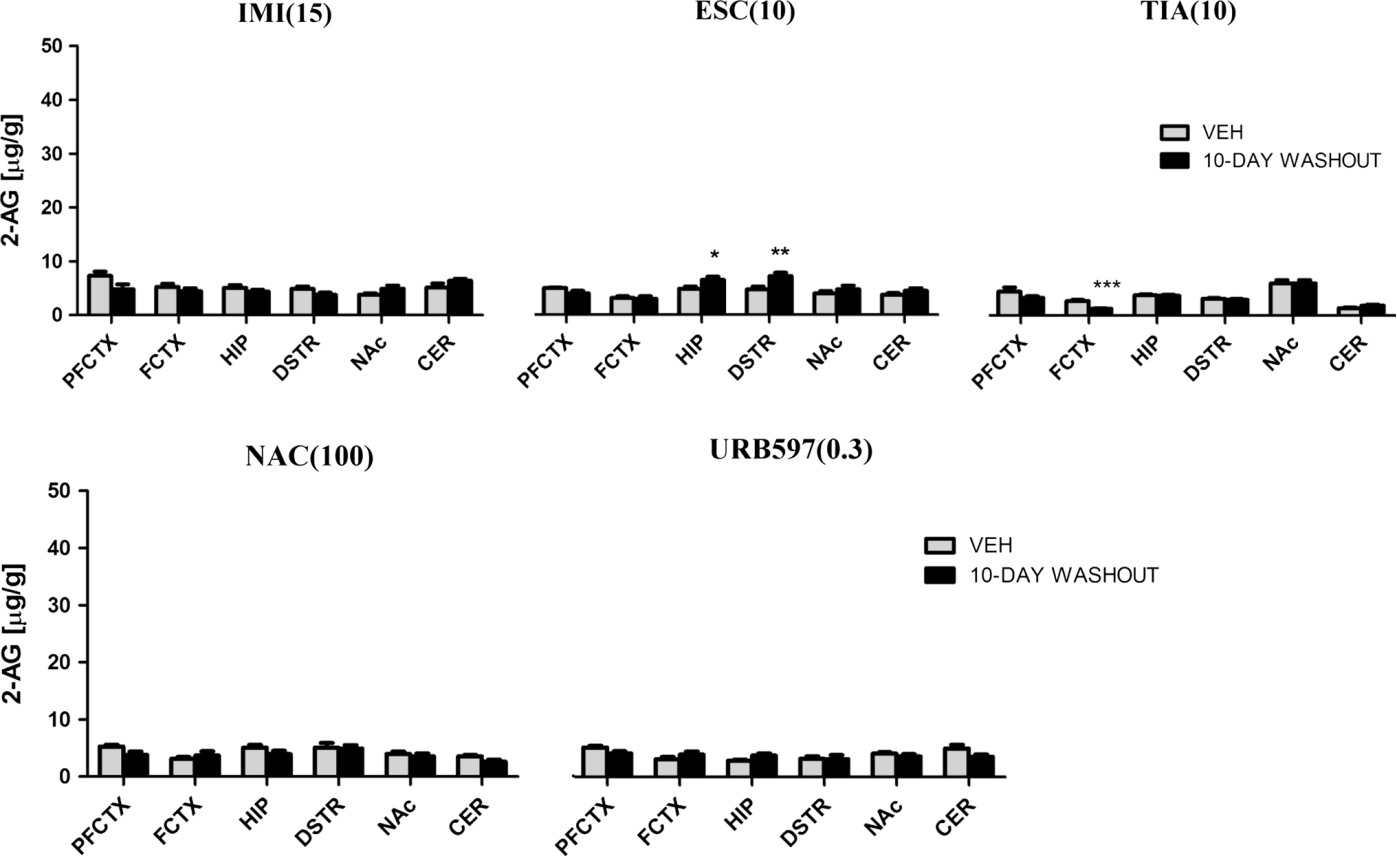
2-AG levels in rat brain structures following chronic drug/compound administration and 10-day washout period. *2-AG* 2-Arachidonoylglycerol, *IMI(15)* imipramine hydrochloride (15 mg/kg), *ESC(10)* escitalopram oxalate, *TIA(10)* tianeptine sodium, *NAC(100)* *N*-acetylcysteine, *URB597(0.3)* cyclohexylcarbamic acid 3-carbamoylbiphenyl-3-yl ester, *PFCTX* prefrontal cortex, *FCTX* frontal cortex, *HIP* hippocampus, *DSTR* dorsal striatum, *NAc* nucleus accumbens, *CER* cerebellum. All data are expressed as the mean ± SEM. *N* = 8 rats/group. *p < 0.05; **p < 0.01; ***p < 0.001 versus corresponding vehicle

IMI (15 mg/kg) treatment resulted in a change in the 2-AG levels in the frontal cortex (*F*(2,21) = 6.385; *p* = 0.0068), dorsal striatum (*F*(2,21) = 11.37; *p* = 0.0005), and cerebellum (*F*(2,21) = 7.035; *p* = 0.0046). The 2-AG levels either increased in the frontal cortex (*p* < 0.05) or decreased in the cerebellum (*p* < 0.05) after acute administration of IMI. IMI administered chronically evoked an increase of 2-AG levels in the frontal cortex (*p* < 0.01) and dorsal striatum (*p* < 0.001), while in the cerebellum (*p* < 0.01) reduced 2-AG levels were reported (Fig. 3). A 10-day washout period after chronic treatment of IMI restored the levels of 2-AG to the levels of vehicle-treated animals in all structures (Fig. 4).

Administration of ESC (10 mg/kg) resulted in potent changes in the 2-AG concentration in the prefrontal cortex (*F*(2,21) = 6.169; *p* = 0.0078), frontal cortex (*F*(2,21) = 8.656; *p* = 0.0018), hippocampus (*F*(2,21) = 3.447; *p* = 0.0508), dorsal striatum (*F*(2,21) = 3.848; *p* = 0.0377), and cerebellum (*F*(2,21) = 3.843; *p* = 0.0378). ESC administered acutely decreased the 2-AG levels in the frontal cortex (*p* < 0.05). Chronic administration of ESC caused a reduction in the 2-AG levels in the prefrontal cortex (*p* < 0.01), frontal cortex (*p* < 0.01), and cerebellum (*p* < 0.05), while an increase of 2-AG concentration was seen in the hippocampus (*p* < 0.05) and dorsal striatum (*p* < 0.05) (Fig. 3). After 10-day drug-free period an increase of 2-AG levels was noted only in the hippocampus (*t* = 2.272, df = 14, *p* < 0.05) and dorsal striatum (*t* = 3.062, df = 14, *p* < 0.01) (Fig. 4).

TIA (10 mg/kg) caused changes in the 2-AG levels in the frontal cortex (*F*(2,21) = 6.997; *p* = 0.0047) and dorsal striatum (*F*(2,21) = 4.073; *p* = 0.032). Repeated daily injections of TIA resulted in an increase of 2-AG levels in the frontal cortex (*p* < 0.01) and dorsal striatum (*p* < 0.05), while TIA administered acutely did not change the 2-AG levels (Fig. 3). 10-day drug-free period caused reduction in the 2-AG levels in the frontal cortex (*t* = 5.294, df = 14, *p* < 0.001) (Fig. 4).

NAC (100 mg/kg) treatment evoked changes in the prefrontal cortex (*F*(2,20) = 9.116; *p* = 0.0015), frontal cortex (*F*(2,21) = 26.09; *p* < 0.0001), dorsal striatum (*F*(2,21) = 22.26; *p* < 0.0001), nucleus accumbens (*F*(2,21) = 8.139; *p* = 0.0024), and cerebellum (*F*(2,21) = 5.187; *p* = 0.0148). NAC administered acutely caused a decrease of 2-AG levels in the prefrontal cortex (*p* < 0.01) and cerebellum (*p* < 0.05). After chronic administration of NAC a decrease of 2-AG concentration was seen in the prefrontal cortex (*p* < 0.05), nucleus accumbens (*p* < 0.01) and cerebellum (*p* < 0.05) and an increase of 2-AG concentration was noted in the frontal cortex (*p* < 0.001) and dorsal striatum (*p* < 0.001) (Fig. 3). A 10-day washout period after chronic treatment of NAC restored the levels of 2-AG to the levels of vehicle-treated animals in all structures (Fig. 4).

Administration of URB597 (0.3 mg/kg) resulted in a change of 2-AG levels only in the dorsal striatum (*F*(2,21) = 4.590; *p* = 0.0222). An increase of 2-AG concentration in the dorsal striatum (*p* < 0.05) was reported after acute or chronic administration of URB597 (Fig. 3). A 10-day washout period after chronic treatment of URB597 restored the levels of 2-AG to the levels of vehicle-treated animals in all structures (Fig. 4).

For comparison, the 2-AG levels did not change 2 h after single administration of URB597 in any structures examined (Table 2).

### Concentration of NAE in Rat Brain Structures

#### PEA

**Fig. 5:**
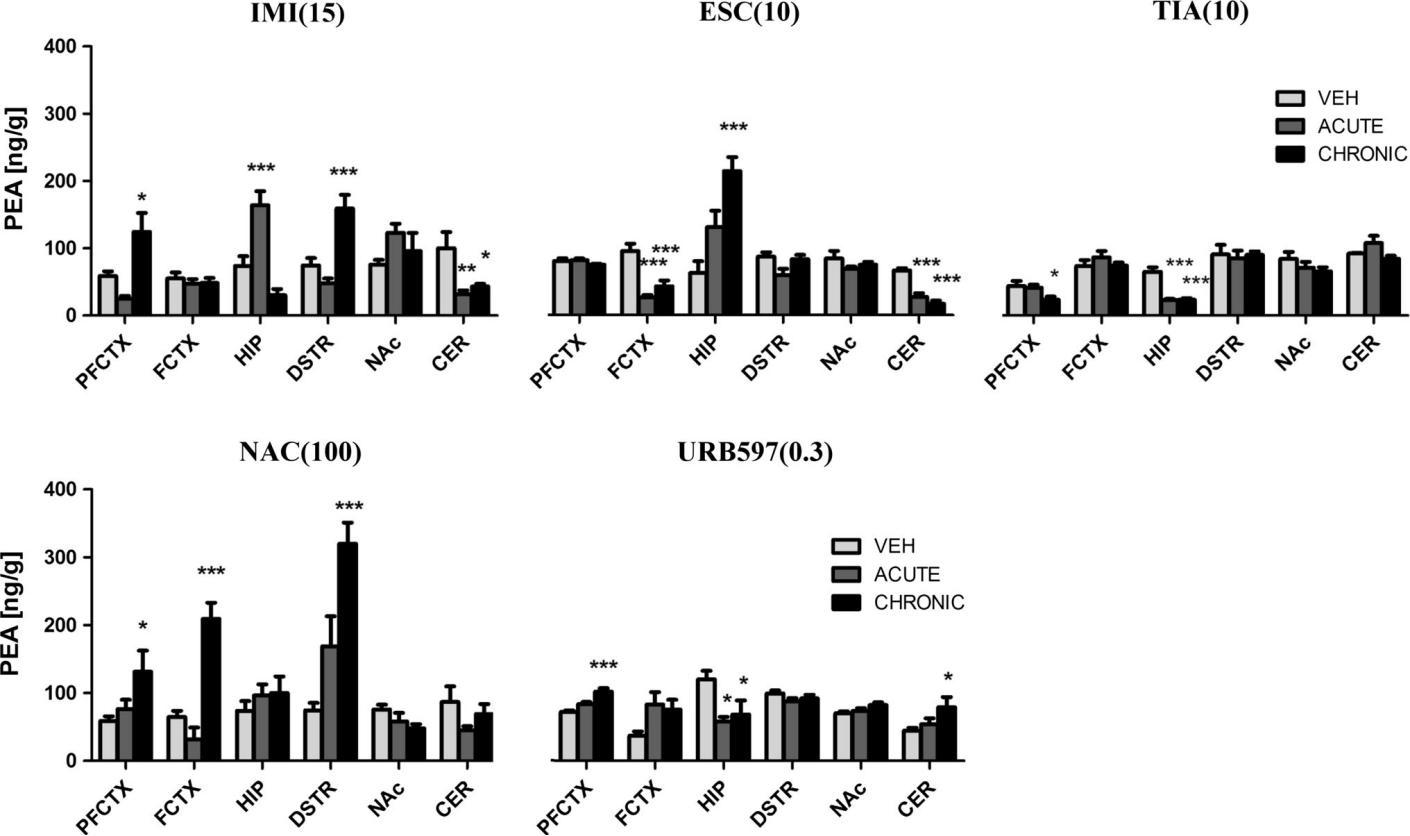
PEA levels in rat brain structures following acute and chronic drug/compound administration. *PEA* Palmitoylethanolamide, *IMI(15)* imipramine hydrochloride (15 mg/kg), *ESC(10)* escitalopram oxalate, *TIA(10)* tianeptine sodium, *NAC(100)* *N*-acetylcysteine, *URB597(0.3)* cyclohexylcarbamic acid 3-carbamoylbiphenyl-3-yl ester, *PFCTX* prefrontal cortex, *FCTX* frontal cortex, *HIP* hippocampus, *DSTR* dorsal striatum, *NAc* nucleus accumbens, *CER* cerebellum. All data are expressed as the mean ± SEM. *N* = 8 rats/group. *p < 0.05; **p < 0.01; ***p < 0.001 versus corresponding vehicle

**Fig. 6:**
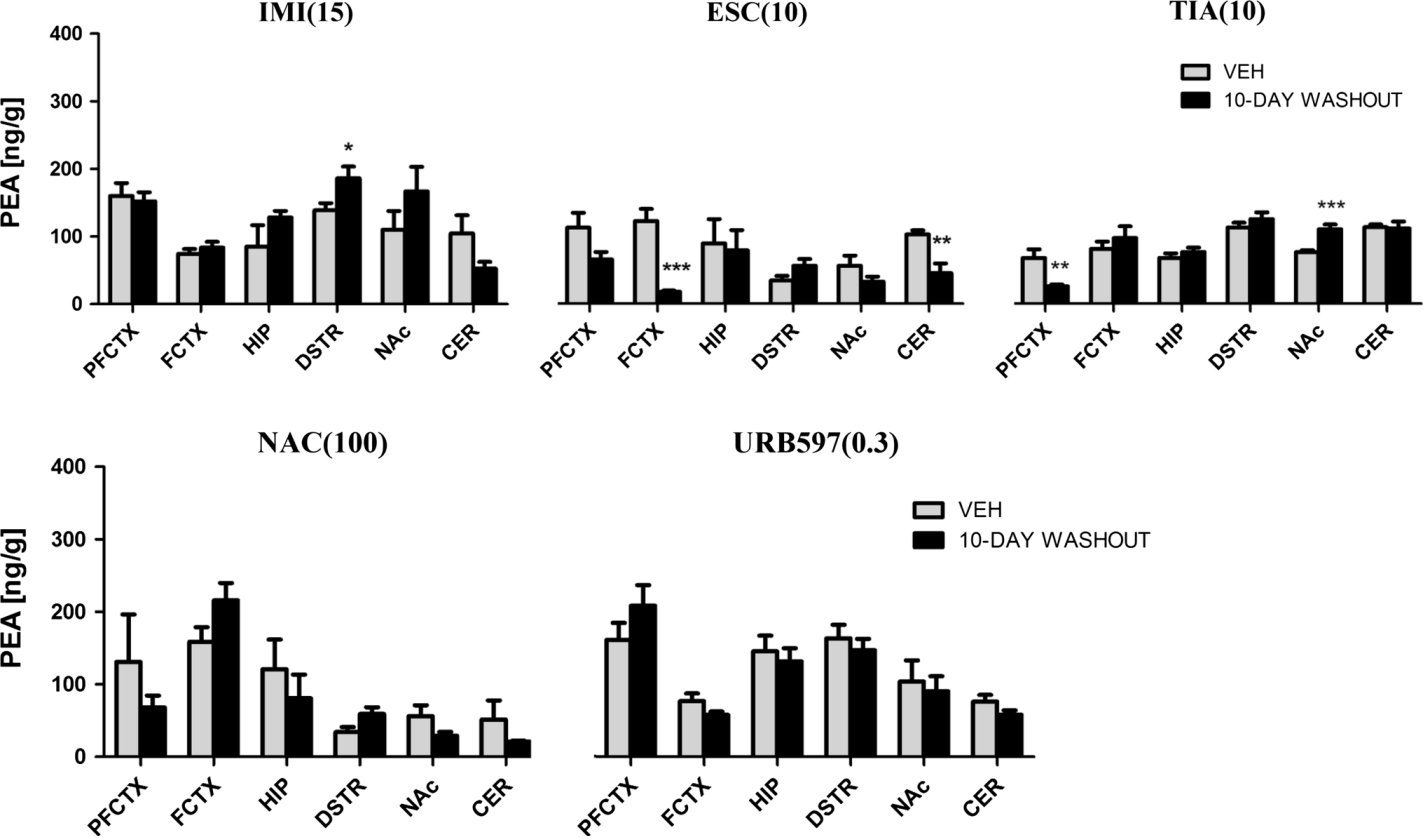
PEA levels in rat brain structures following chronic drug/compound administration and 10-day washout period. *PEA* Palmitoylethanolamide, *IMI(15)* imipramine hydrochloride (15 mg/kg), *ESC(10)* escitalopram oxalate, *TIA(10)* tianeptine sodium, *NAC(100)* *N*-acetylcysteine, *URB597(0.3)* cyclohexylcarbamic acid 3-carbamoylbiphenyl-3-yl ester, *PFCTX* prefrontal cortex, *FCTX* frontal cortex, *HIP* hippocampus, *DSTR* dorsal striatum, *NAc* nucleus accumbens, *CER* cerebellum. All data are expressed as the mean ± SEM. *N* = 8 rats/group. *p < 0.05; **p < 0.01; ***p < 0.001 versus corresponding vehicle

IMI (15 mg/kg) treatment resulted in changes of the PEA concentration in the prefrontal cortex (*F*(2,20) = 10.48; *p* = 0.0008), hippocampus (*F*(2,21) = 19.65; *p* < 0.0001), dorsal striatum (*F*(2,21) = 16.98; *p* < 0.0001), and cerebellum (*F*(2,21) = 6.512; *p* = 0.0063). After acute administration of IMI we observed either a decrease (in the cerebellum (*p* < 0.01)) or an increase of PEA concentration (in the hippocampus (*p* < 0.001)). An increase of PEA levels was seen in the prefrontal cortex (*p* < 0.05) and dorsal striatum (*p* < 0.001), and a decrease was seen in cerebellum (*p* < 0.05) after chronic administration of IMI (Fig. 5). An increase of PEA levels was observed in the dorsal striatum (*t* = 2.328, df = 14, *p* < 0.05) 10 days after the last administration of IMI (Fig. 6).

After ESC (10 mg/kg) treatment, we noted that the PEA levels changed in the frontal cortex (*F*(2,21) = 18.56; *p* < 0.0001), hippocampus (*F*(2,21) = 12.98; *p* = 0.0002) and cerebellum (*F*(2,21) = 35.23; *p* < 0.0001). A potent decrease of PEA levels was seen in the frontal cortex (*p* < 0.001) and cerebellum (*p* < 0.001) after acute administration of ESC. ESC administrated chronically caused an increase of PEA levels in the hippocampus (*p* < 0.001) and a decrease of PEA levels in the frontal cortex (*p* < 0.001) and cerebellum (*p* < 0.001) (Fig. 5). A 10-day washout period resulted in reduction of PEA concentration in the frontal cortex (*t* = 5.744, df = 14, *p* < 0.001) and cerebellum (*t* = 3.683, df = 14, *p* < 0.01) (Fig. 6).

Administration of TIA (10 mg/kg) caused changes in the PEA levels in the prefrontal cortex (*F*(2,21) = 3.558; *p* = 0.0477) and hippocampus (*F*(2,21) = 36.07; *p* < 0.0001). A potent decrease of PEA levels was seen in the hippocampus (*p* < 0.001) after acute administration of TIA. TIA administrated chronically caused a decrease of PEA levels in the prefrontal cortex (*p* < 0.05) and hippocampus (*p* < 0.001) (Fig. 5). A 10-day drug-free period resulted in reduction of PEA concentration in the prefrontal cortex (*t* = 3.111, df = 14, *p* < 0.01) and an increase of PEA levels was reported in the nucleus accumbens (*t* = 4.432, df = 14, *p* < 0.001) (Fig. 6).

NAC (100 mg/kg) caused changes in the PEA levels in the cortical structures (prefrontal (*F*(2,20) = 3.954; *p* = 0.0357) and frontal cortex (*F*(2,21) = 28.12; *p* < 0.0001)) and dorsal striatum (*F*(2,21) = 15.10; *p* < 0.0001). Repeated daily injections of NAC resulted in an increase of PEA levels in the prefrontal cortex (*p* < 0.05), frontal cortex (*p* < 0.001), and dorsal striatum (*p* < 0.001), while NAC administered acutely did not change the PEA levels (Fig. 5). A 10-day washout period after chronic treatment of NAC restored the levels of PEA to the levels of vehicle-treated animals in all structures (Fig. 6).

Administration of URB597 (0.3 mg/kg) resulted in changes in the PEA concentration in the prefrontal cortex (*F*(2,21) = 16.21; *p* < 0.0001), hippocampus (*F*(2,21) = 5.364, *p* = 0.0131), and cerebellum (*F*(2,21) = 3.054; *p* = 0.0685). URB597 administered acutely decreased the PEA levels in the hippocampus (*p* < 0.05). Chronic administration of URB597 caused a reduction in the PEA levels in the hippocampus (*p* < 0.05), while an increase of PEA concentration was seen in the prefrontal cortex (*p* < 0.001) and cerebellum (*p* < 0.05) (Fig. 5). A 10-day washout period after chronic treatment of URB597 restored the levels of PEA to the levels of vehicle-treated animals in all structures (Fig. 6).

For comparison, the levels of PEA measured 2 h after single administration of URB597 increased in the hippocampus (*t* = 3.436, df = 10, *p* < 0.01), dorsal striatum (*t* = 5.444, df = 10, *p* < 0.001), and nucleus accumbens (*t* = 7.998, df = 10, *p* < 0.001) (Table 2).

#### OEA

**Fig. 7:**
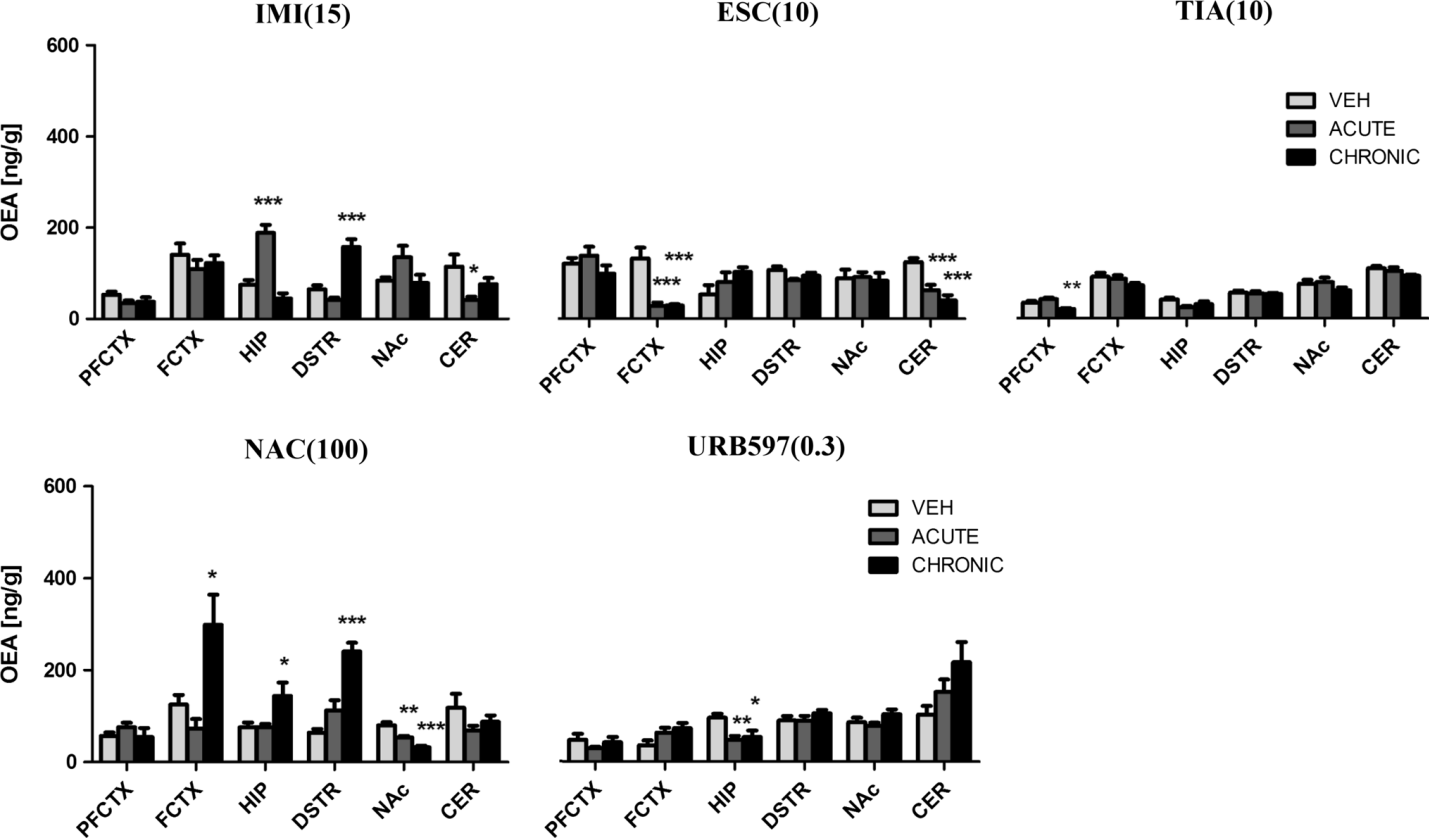
OEA levels in rat brain structures following acute and chronic drug/compound administration. *OEA* Oleoylethanolamide, *IMI(15)* imipramine hydrochloride (15 mg/kg), *ESC(10)* escitalopram oxalate, *TIA(10)* tianeptine sodium, *NAC(100)* *N*-acetylcysteine, *URB597(0.3)* cyclohexylcarbamic acid 3-carbamoylbiphenyl-3-yl ester, *PFCTX* prefrontal cortex, *FCTX* frontal cortex, *HIP* hippocampus, *DSTR* dorsal striatum, *NAc* nucleus accumbens, *CER* cerebellum. All data are expressed as the mean ± SEM. *N* = 8 rats/group. *p < 0.05; **p < 0.01; ***p < 0.001 versus corresponding vehicle

**Fig. 8:**
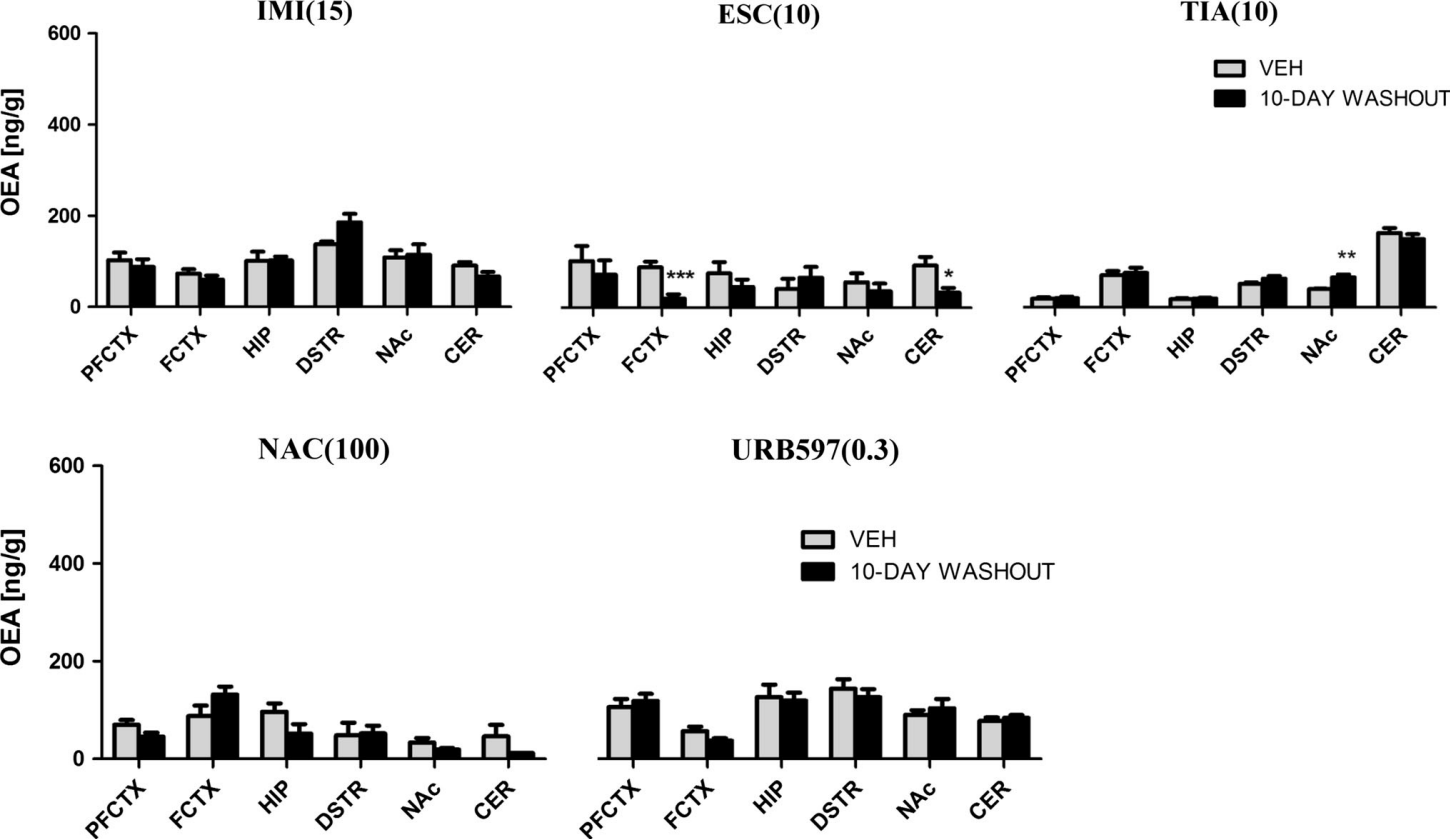
OEA levels in rat brain structures following chronic drug/compound administration and 10-day washout period. *OEA* Oleoylethanolamide, *IMI(15)* imipramine hydrochloride (15 mg/kg), *ESC(10)* escitalopram oxalate, *TIA(10)* tianeptine sodium, *NAC(100)* *N*-acetylcysteine, *URB597(0.3)* cyclohexylcarbamic acid 3-carbamoylbiphenyl-3-yl ester, *PFCTX* prefrontal cortex, *FCTX* frontal cortex, *HIP* hippocampus, *DSTR* dorsal striatum, *NAc* nucleus accumbens, *CER* cerebellum. All data are expressed as the mean ± SEM. *N* = 8 rats/group. *p < 0.05; **p < 0.01; ***p < 0.001 versus corresponding vehicle

After administration of IMI (15 mg/kg), we observed changes in the OEA concentration in the hippocampus (*F*(2,21) = 31.62; *p* < 0.0001), dorsal striatum (*F*(2,21) = 28.73; *p* < 0.0001), and cerebellum (*F*(2,21) = 4.33; *p* = 0.0266). IMI administered acutely increased the OEA levels in the hippocampus (*p* < 0.001) and decreased the OEA levels in the cerebellum (*p* < 0.05). Chronic administration of IMI caused an increase of OEA concentration in the dorsal striatum (*p* < 0.001) (Fig. 7). A 10-day washout period after chronic treatment of IMI restored the levels of OEA to the levels of vehicle-treated animals in all structures (Fig. 8).

ESC (10 mg/kg) caused changes in the OEA levels in the frontal cortex (*F*(2,21) = 17.65; *p* < 0.001) and cerebellum (*F*(2,21) = 17.25; *p* < 0.0001). A decrease of basal levels of OEA was observed in the frontal cortex (*p* < 0.001) and cerebellum (*p* < 0.001) after acute and chronic administration of ESC (Fig. 7). 10-day washout period caused reduction in the OEA levels in the frontal cortex (*t* = 4.305, df = 14, *p* < 0.001) and cerebellum (*t* = 2.720, df = 14, *p* < 0.05) (Fig. 8).

TIA (10 mg/kg) treatment caused changes in the OEA levels only in the prefrontal cortex (*F*(2,21) = 12.38; *p* = 0.0003). A significant decrease was observed in the prefrontal cortex (*p* < 0.01) after chronic administration of TIA, while TIA administered acutely did not change the OEA levels (Fig. 7). 10-day drug-free period caused an increase of the OEA levels in the nucleus accumbens (*t* = 3.881, df = 14, *p* < 0.01) (Fig. 8).

After NAC (100 mg/kg) administration we observed changes in the OEA levels in the frontal cortex (*F*(2,21) = 8.198; *p* = 0.0023), hippocampus (*F*(2,21) = 4.576; *p* = 0.0224), dorsal striatum (*F*(2,21) = 27.42; *p* < 0.0001) and nucleus accumbens (*F*(2,20) = 25.95; *p* < 0.0001). A significant decrease of OEA concentration was noted in the nucleus accumbens (*p* < 0.01) after acute administration of NAC. After chronic administration of NAC the OEA levels either decreased (in the nucleus accumbens (*p* < 0.001)) or increased (in the frontal cortex (*p* < 0.05), hippocampus (*p* < 0.05) and dorsal striatum (*p* < 0.001)) (Fig. 7). A 10-day washout period after chronic treatment of NAC restored the levels of OEA to the levels of vehicle-treated animals in all structures (Fig. 8). URB597 (0.3 mg/kg) treatment resulted in a change of OEA levels only in the hippocampus (*F*(2,21) = 6.032; *p* = 0.0085). The OEA levels decreased in the hippocampus after single and chronic administration of URB597 (*p* < 0.01 and *p* < 0.05, respectively) (Fig. 7). A 10-day washout period after chronic treatment of URB597 restored the levels of OEA to the levels of vehicle-treated animals in all structures (Fig. 8).

For comparison, the levels of OEA measured 2 h after single administration of URB597 increased in the hippocampus (*t* = 2.686, df = 10, *p* < 0.05), dorsal striatum (*t* = 4.740, df = 10, *p* < 0.001), and nucleus accumbens (*t* = 4.305, df = 10, *p* < 0.01) (Table 2).

## Discussion

This paper reveals the effects of both antidepressants and drugs with antidepressant-like activity (see “Introduction” section) on the levels of eCBs and NAEs in *ex vivo* tissue. We examined several brain structures that are either implicated in the pathogenesis of depression (i.e., the prefrontal cortex, frontal cortex, and hippocampus) (Holmes 2008) or linked to anhedonia (i.e., the striatal areas) (Robinson et al. 2012) and are sites of biochemical and morphological changes in depressed patients (Holmes 2008). Additionally, the cerebellum has been recently identified as an area that receives negative functional connectivity from the hippocampus in depressed subjects (Cao et al. 2012). Our results suggest that chronic treatment with antidepressants results in higher levels of AEA in the hippocampus and dorsal striatum along with increased levels of 2-AG in the dorsal striatum. These changes were even maintained after a 10-day drug-free period that followed repeated treatment with ESC and TIA. This is the first study to report alterations in the levels of eCBs and NAEs in the brain after the administration of clinically approved antidepressant drugs (IMI, ESC, and TIA) or drugs with antidepressant-like activity (NAC and URB597).

Some changes in eCBs/NAEs levels could even be observed only 24 h after a single dose the tested drugs. For example, a single dose of either IMI or NAC evoked a significant increase in AEA levels in the hippocampus or dorsal striatum, respectively. Additionally, a single dose of IMI or URB597 increased the levels of 2-AG in the frontal cortex and dorsal striatum, respectively. In contrast, a single dose of either IMI or NAC decreased 2-AG levels in the cerebellum, while ESC and NAC have a similar effect on cortical structures. Administering a single dose of TIA or URB597 resulted in a significant decrease in NAE levels in the hippocampus (PEA and PEA/OEA, respectively), while a single dose of IMI had the opposite effect in this region. Additionally, NAC decreased NAE (OEA) levels in the nucleus accumbens, and ESC decreased NAE levels (both PEA/OEA) in both the frontal cortex and the cerebellum. These changes occurred even though the drugs were rapidly eliminated and both eCBs and NAEs were rapidly degraded. These results imply that acute drug administration can provoke rapid adaptive changes that begin only 24 h after a single dose. Interestingly, these changes were all maintained after chronic administration of these drugs over the course of 14 days with the exception of the increase in hippocampal NAE levels that was observed after a single dose of IMI. Finally, the adaptive changes in the frontal cortex and cerebellum that followed ESC treatment were maintained even after a 10-day ESC-free period.

A potent rise in the levels of eCBs, AEA and 2-AG, was observed in the rat dorsal striatum 24 h after the chronic administration of all tested drugs. In the present paper we also report that striatal eCB levels also increase in response to repeated URB597 treatment. Additionally, withdrawal of this drug for 24 h initiates adaptive changes within the eCB system, which may be associated with the antidepressant-like activity of this FAAH inhibitor. Injecting URB597 2 h before decapitation induced a potent increase in the levels of AEA, PEA, and OEA in multiple brain structures, possibly because it acts in time-dependent manner in which an increase of AEA levels lasts between 30 min and 2 h while PEA/OEA levels are maintained up to 6 h (the present paper; Kathuria et al. 2003; Fegley et al. 2005; Piomelli et al. 2006). A previously study by Bortolato et al. (2007) has suggested that treatment for 5 weeks with URB597 also enhances striatal AEA levels but does not affect 2-AG levels in control rats or rats exposed to chronic mild stress (CMS) (Bortolato et al. 2007). Our findings suggest that the antidepressant drugs may exert their therapeutic effects by normalizing eCB levels within the striatum that have been disturbed during depression. In support of this hypothesis, one cortical symptom of depression is anhedonia, which has been linked to the abnormal functioning of CB_1_ receptors in the ventral striatum in rats (Hill et al. 2008b). These same alterations have also been observed in anhedonia-related animal models of depression, including chronic unpredictable stress (CUS) and CMS (Hill et al. 2008b; Reich et al. 2013a, b; Segev et al. 2013). Anhedonia is associated with a weakening of the eCB signal in the ventral striatum and with reduced local levels of AEA (Hill et al. 2008b). In this study we detected changes in eCB levels in the dorsal striatum in response to treatment with IMI, ESC, TIA, NAC, or URB597. In contrast, eCB levels only changed in the ventral region (the nucleus accumbens) after chronic administration of NAC. It is still unclear whether changes in eCB levels directly altered the levels of CB receptors or enzymes, although one previous report indicated that an increase in the density of CB_1_ receptors was observed in the ventral striatum after reduced levels of AEA (via increased FAAH activity) occurred in alcoholic suicide victims (Vinod et al. 2010). In this paper, we also report that striatal NAE levels increased after chronic treatment with IMI and NAC. One possibility is that increased PEA and OEA levels could strengthen the effect of AEA on CB or vanilloid (TRPV1) receptors (i.e., the “entourage effect”), which could in turn potentiate the effect of eCBs (De Petrocellis et al. 2001; Smart et al. 2002). Another possibility is that NAEs may increase hippocampal ceramide levels, stabilize mitochondrial function and inhibit the degradation of AEA, which could together have a neuroprotective effect (Skaper et al. 1996; Nagayama et al. 1999). Our findings add to the previous scientific literature regarding the effects of antidepressants on the eCB system with one important contradiction. IMI was previously found to lower the expression of CB_1_ receptors in the ventral striatum (Hill et al. 2008b) but had no effect on eCB levels in the rat brain (Bortolato et al. 2007; Hill et al. 2008b). The rise in eCB levels that we observed in this study may be result of either differing IMI dosages (Hill et al. used a lower dose), duration of treatment (Bortolato et al. used a 5-week procedure), and/or the sensitivity of the different methods used to measure eCB levels (isotope-dilution liquid chromatography|[minus]|mass spectrometry vs. LC–MS/MS).

As in the striatum, chronic treatment with antidepressant drugs also enhances AEA levels in the hippocampus. Previous studies have demonstrated that eCB signaling decreases in the hippocampus in an animal model of depression (Hill et al. 2005). Additionally, a reduction in hippocampal size has been observed during depression (McLaughlin and Gobbi 2012), and the local administration of a CB1 receptor agonist in the dentate gyrus has elicited an antidepressant-like response (McLaughlin et al. 2007). The eCB system, particularly AEA/CB_1_ receptor signaling, is an important mediator of neurogenesis within the hippocampus. The activation of these receptors directs neural precursor cells into a mitogenic state via the activation of the phosphatidylinositol-3 kinase (PI3K)/Akt pathway, which promotes cannabinoid-induced proliferation (Galve-Roperh et al. 2002; Ozaita et al. 2007). Increased hippocampal levels of AEA can both reduce excitotoxic damage within the hippocampus and induce protective mechanisms in hippocampal neurons, which may be linked to the influence of the eCB system on the hypothalamic–pituitary–adrenal (HPA) axis (Marsicano et al. 2003). Thus, rats subjected to chronic stress or repeated administration of corticosteroids experience a drop in both the concentration of eCBs and expression of CB_1_ receptors in the hippocampus (Hill et al. 2005, 2008a, 2009). Furthermore, activation of the stress axis, which results from a reduction in the inhibitory effect of hippocampal neurons on limbic structures, has been associated with decreased activation of the eCB system in local GABAergic neurons (Hu et al. 2011). At sites of GABAergic inputs in the hippocampus (CA1 region), the activation of CB_1_ receptors induces various mechanisms of synaptic plasticity, including depolarization-induced suppression of inhibition (DSI) and long-term depression of inhibitory synapses (I-LTD) (Lovinger 2008). Additionally, previous studies have suggested that hippocampal levels of 2-AG are elevated 24 h or 10 days after chronic administration of ESC. A recent study found that inhibiting monoacylglycerol lipase (MAGL), which is an enzyme involved in 2-AG degradation, produces antidepressant-like effects through the enhancement of eCB signaling through the mammalian target of rapamycin (mTOR) pathway in the hippocampus (Zhong et al. 2014), which suggests a possible involvement of increased 2-AG levels in the antidepressant mechanism of ESC. In addition to eCBs, NAE levels also change in the rat hippocampus. IMI elicits an increase in both PEA and OEA, while ESC increases PEA levels and NAC increases OEA levels. In contrast, TIA decrease PEA levels, and URB597 decreases both PEA and OEA levels. Along with eCBs, these NAEs may also participate in controlling synaptic plasticity via Kv4.3 potassium channels in hippocampal interneurons along with ascending pyramidal and GABAergic cortical neurons (Burkhalter et al. 2006; Bourdeau et al. 2007). As reported previously, chronic treatment with desipramine (a NA and 5-HT reuptake inhibitor) or tranylcypromine (a monoamine oxidase inhibitor) enhances the expression of CB_1_ receptors in the hippocampus, although only tranylcypromine decreased AEA levels in the hippocampus (Hill et al. 2006, 2008c). These studies suggest that the regulation of CB_1_ receptors in specific brain structures after antidepressant treatment might result from adaptive changes and could vary depending on the levels of both receptors and ligands. In particular, Bortolato et al. suggested that chronic treatment with URB597 did not increase hippocampal AEA levels; in fact, prolonged (5 week) exposure may instead down-regulate AEA in the hippocampus (Bortolato et al. 2007). However, this effect is still poorly understood.

As reported, there were significant alterations in eCB and NAE levels the rat prefrontal cortex, which participates in a variety of functions including learning and memory. For example, increased activation of the eCB system has been observed to strengthen memory (Lafourcade et al. 2007). Reinforcing emotional memories of aversive stimuli can increase levels of eCBs in the prefrontal cortex, which may induce emotional discomfort during depression. In fact, elevated levels of eCBs and CB_1_ receptors have been observed in the dorsolateral prefrontal cortex of alcoholic suicide victims (Vinod et al. 2005). Here, we observed a decrease in the concentration of 2-AG after the chronic administration of ESC and NAC, which may be a potential mechanism for the antidepressant-like activity of these drugs in the prefrontal cortex. In contrast, Hill et al. (2008c) indicated that tranylcypromine increases the level of 2-AG and enhances the density of CB_1_ receptors in the prefrontal cortex (Hill et al. 2008c). However, other reports have demonstrated that CB_1_ receptors in the prefrontal cortex can participate in the antidepressant actions of CB_1_ receptor agonists and that increases in local AEA signaling can modulate stress coping behaviors via the activation of serotonergic neurons in the raphe nucleus (Bambico et al. 2007; McLaughlin et al. 2012). Additionally, chronically administering fluoxetine can fully reverse the enhanced CB_1_-receptor signaling seen in bulbectomized rats (Rodriguez-Gaztelumendi et al. 2009) and can also modulate the function (but not the density) of CB_1_ receptors in the prefrontal cortex (Mato et al. 2010). In this study, we also noted an increase in PEA levels in the prefrontal cortex after chronic treatment with IMI, NAC and the FAAH inhibitor. Because PEA administration (5–40 mg/kg) by itself can reduce a mouse’s immobility time in the TST and FST, which is a behavioral indication of antidepressant-like activity (Yu et al. 2011), the increased PEA levels might contribute to the antidepressant-like effect these drugs. In contrast, a decrease in NAE levels was observed in the prefrontal cortex after TIA chronic treatment, and reduced PEA levels were maintained after the TIA-free period. This TIA-specific effect might be related to the adaptive changes associated with NAE depletion or to changes in eCB receptors, enzymes or transporters.

Anti-depressant drugs had different effects in other brain regions. In the frontal cortex, chronic administration of NAC increased AEA levels, while 2-AG levels increased after IMI, TIA and NAC treatment but decreased after ESC treatment. Changes in cortical eCB levels has not yet been established in postmortem or *ex vivo* studies, although it has been observed that CB_1_ receptor density decreases in mood disorders (Koethe et al. 2007) and increases in both depressed suicide victims and animal models of depression (Hungund et al. 2004; Choi et al. 2012). Long-term fluoxetine treatment in obese Zucker rats reduces these elevated CB_1_ receptor levels in the frontal cortex, which suggests that the eCB system is involved in mediating the effects of fluoxetine via the influence of 5-HT enhancement on CB_1_ receptor levels (Zarate et al. 2008). NAE levels in the frontal cortex also fell both 24 h and 10 days after ESC treatment was withdrawn, which many contribute to the antidepressant effect of ESC via the dampening TRPV1-mediated signaling. In support of this hypothesis, previous studies have suggested that the loss of TRPV1 results in antidepressant, anxiolytic, abnormal social and reduced memorial behaviors (You et al. 2012). However, the exact mechanism remains unclear.

In contrast to chronic IMI treatment, cortical NAE levels were reduced after treatment with TIA and ESC, which most likely stems from their differential effects on NA and 5-HT signaling. One possibility is that the increase in NAE levels observed after IMI treatment might reduce NA release and normalize the increased synaptic availability that is induced by IMI treatment; however, future studies are needed to test this hypothesis.

We also examined the effect of chronic antidepressant treatment on the rat cerebellum, which has recently been implicated in the pathogenesis of depression, specifically disturbances in cerebellar–hippocampal projections (Cao et al. 2012). In this study, we report drug-dependent changes in cerebellar levels of both eCBs (AEA increases after the chronic administration of URB597, while 2-AG decreases after the acute or chronic administration of IMI and NAC and the chronic administration of ESC) and NAEs (PEA increases after the chronic administration of URB597 but PEA and OEA decrease after chronic treatment with IMI or ESC). eCBs act as retrograde messengers in the cerebellum, which allows eCB signals to be transmitted through depolarization of Purkinje cells or local interneurons and permits signal transmission over long distances (Kreitzer et al. 2002). Suarez et al. (2008) detected the presence of components of the eCB system in cerebellar tissue, which suggests that eCBs might participate in the development of cerebellar synaptic plasticity [either long term depression (LTD) or long term potentiation (LTP)] (Suarez et al. 2008). Lowered levels of 2-AG after antidepressant treatment (IMI, ESC and NAC) might regulate the plasticity of synapses being made onto Purkinje cells and could play a key role in normalizing LTD in the cerebellar cortex (Safo et al. 2006; Carey et al. 2011; Zhong et al. 2011).

Interestingly, the effects of antidepressants on the eCB system seem to be short-lived. After a 10-day washout period, eCB concentrations returned to control (vehicle) levels except in animals treated with ESC and TIA. The chronic administration of ESC altered eCB levels in multiple brain regions (e.g., frontal cortex, hippocampus, dorsal striatum, and cerebellum), and these effects were maintained even after the drug-free period. It is still unclear whether adaptive changes existed within the eCB system (e.g., changes in enzyme activity, receptor density, eCB transport, etc.) after 14 days of ESC treatment. However, the drug-free period did increase the levels of NAEs in the nucleus accumbens, which was not observed after the acute or chronic administration of TIA. TIA possesses a unique mechanism of antidepressive action and has a specific pharmacokinetic profile. In fact, recent studies have established that unlike other antidepressants, TIA enhances serotonin reuptake and is not primarily metabolized by the hepatic cytochrome P450 system. TIA also stimulates DA release in the nucleus accumbens and acts as a glutamatergic modulator, which influences central neuronal remodeling and restoration of neuronal plasticity (Invernizzi et al. 1992; Vadachkoria et al. 2009). However, understanding the relevance of these multi-targeted interactions will require further study.

## Conclusion

As demonstrated here, changes in eCB and NAE levels in various rat brain structures indicate that these lipids may play a significant role in the mechanism of the anti-depressant drugs IMI, ESC, TIA, NAC, and URB597. Further studies involving selective antagonists of CB_1_ and CB_2_ receptors will be necessary to accurately determine the role of the eCB system in the mechanism of action of these drugs. Together, these studies will help determine whether the stimulation of a specific cannabinoid receptor is necessary or if increased eCB action on other targets (e.g., TPRV1 or PPARα) is instead responsible. Further studies are also needed to explore whether the eCB system could function as a “central player” in the pathogenesis of depression through its effects on cellular processes (levels of neurotransmitters, processes of neurogenesis, HPA axis, etc.). Understanding the role of the eCB system in the mechanism of action of clinically effective antidepressants may implicate eCBs as a target for drug design and discovery.

## Abbreviations

AEA: Anandamide
2-AG: 2-Arachidonoylglycerol
eCBs: Endocannabinoids
ESC: Escitalopram
IMI: Imipramine
LC–MS/MS: Liquid chromatography tandem mass spectrometry
NAC: *N*-Acetylcysteine
NAEs: *N*-Acylethanolamines
OEA: Oleoylethanolamide
PEA: Palmitoylethanolamide
TIA: Tianeptine
